# A Decolonial Lexicon for Immunology

**DOI:** 10.1007/s10441-026-09527-6

**Published:** 2026-05-09

**Authors:** Claudio Vieira da Silva

**Affiliations:** Universidade Federal de Uberlândia, Uberlândia, Minas Gerais Brasil

**Keywords:** Immunology, Decolonization, Medical humanities, Metaphor, Holobiont, Self/non-self, Philosophy of biology, Relationality

## Abstract

The discipline of immunology has historically been foundationally framed by metaphors of war, portraying the body as a sovereign state defending its territory against foreign invaders. This paradigm, however, is not strictly a biological necessity but also a historical artifact of colonial logic that arguably limits our understanding of symbiosis, tolerance, and the nature of relational pathologies. This paper argues for a decolonial paradigm shift, proposing a comprehensive reframing of the immune system not as a military force but as a sophisticated system of communication, governance, and diplomacy within a multi-species community, the holobiont. We trace the colonial genealogy of war metaphors and expose its conceptual inadequacies in the face of modern biology, distinguishing between historical rhetorical resonances and causal scientific developments. Drawing inspiration from relational and ecological philosophies, we then propose a new conceptual lexicon, using the metaphor of the body as a quilombo, a diverse and resilient community. By separating canonical biological mechanisms from metaphorical interpretation, this framework reframes core immunological processes: inflammation becomes an urgent community assembly, the adaptive response a journey of information, and pathologies like autoimmunity, cancer, and immunodeficiency become crises of communication and social cohesion. Offered as a conceptual heuristic rather than a wholesale structural equivalent, this relational approach offers not only new avenues for research and therapy but also serves as a powerful pedagogical tool to foster a more holistic, integrated, and ecologically conscious view of life itself.

## Introduction

### The Invisible Scaffolding of War

The language of immunology is a language of war. From the first pages of foundational textbooks to the pronouncements of clinicians and the headlines of popular science, the story is one of conflict (Abbas et al. 2019; Murphy and Weaver 2017). Our bodies are sovereign territories, the self, under perpetual siege from a world of non-self-others. Our immune system is a national defense force, a complex military apparatus with a clear mandate: seek and destroy. It deploys armies of cells, neutrophil shock troops, macrophage generals, and lymphocyte special forces from lymphoid barracks to patrol our borders and annihilate invaders. Molecular weapons are fired, killer cells execute their targets, and a state of ceaseless vigilance is maintained. We fight off a cold, battle cancer, and develop drugs that boost our defenses (Martin 1994).

This narrative is undeniably powerful. It provides a simple, intuitive, and compelling framework for a dizzyingly complex biological system. Yet, as Susan Sontag warned in Illness as Metaphor, such metaphors are never innocent (Sontag 2001). They are not descriptive but prescriptive; they structure our understanding, guide our research, and shape our therapeutic interventions. As George Lakoff and Mark Johnson argued, the metaphors we live by create realities (Lakoff and Johnson 1980). The war metaphor in immunology is so deeply entrenched that it is often mistaken for an objective description of nature. It has become an invisible scaffolding, a conceptual framework that dictates the questions we ask and the answers we are capable of hearing.

This paper argues that the war metaphor is a historical and political artifact, not a strict biological necessity, serving as a heuristic that both illuminates and obscures. Its logic is a direct inheritance of a specific worldview, consolidated at the apex of European imperialism and rooted in the Westphalian concept of the nation-state (Tauber 2017). It is a colonial logic, projecting the dynamics of conquest, purification, and border control onto the most intimate processes of life (Quijano 2000). While this framework offered an effective narrative in the era of germ theory, it is profoundly ill-equipped to handle the conceptual revolutions of 21st-century biology, most notably the discovery of the holobiont, the recognition that we are not singular individuals but complex, multi-species ecosystems (Gilbert et al. 2012; Yong 2016).

The military paradigm forces us to view fundamental biological processes through a distorted lens. Symbiosis with our microbiota becomes a tense occupation. Tolerance, the default state of non-reactivity, appears to be a paradoxical failure to attack. Pathology is invariably framed as a military error: autoimmunity is a civil war, allergies are a disproportionate response, and cancer is an internal insurrection. This language is not only biologically misleading but also fosters an adversarial relationship with our own bodies and a medical practice focused on annihilation rather than the restoration of balance.

A decolonial turn in immunology is therefore not just a semantic exercise; it is a conceptual re-evaluation. To be clear, this paper does not claim that scientific mechanisms are factually incorrect, but rather that our interpretive models shape how we prioritize, teach, and engage with these mechanisms. It requires dismantling the colonial scaffolding of war and building a new one based on relationality, communication, and ecology. This paper proposes such an alternative as a contribution to an ongoing conversation within the philosophy of biology. I advocate for a paradigm shift from the body as a battlefield to the body as a community. Specifically, I offer the metaphor of the body as a quilombo, a term from Brazilian history for a community formed by escaped slaves and other marginalized groups, a space defined by its resilience, diversity, and complex internal relationships (Krenak 2019).

In this new framework, the immune system is not a military but a system of governance and communication. Its purpose is not defense but the maintenance of dynamic equilibrium within a vibrant, multi-species community. This paper will first trace the colonial genealogy of war metaphors. Second, it will expose its conceptual limitations. Third, it will systematically build a new, relational lexicon, explicitly separating standard biological mechanisms from their metaphorical interpretations, re-describing the anatomy and processes of the immune system in the language of civic life. Finally, it will explore the profound pedagogical, therapeutic, and ethical implications of seeing our bodies not as fortresses under siege, but as worlds in constant, vital conversation (Viveiros de Castro 2017).

### The Colonial Genealogy of the Immune Self

The language of immunology did not emerge in a vacuum. Its foundational concepts were forged in the intellectual and political crucible of the late 19th and early 20th centuries, a period dominated by three intersecting forces: the rise of germ theory, the consolidation of the European nation-state, and the zenith of global imperialism (Tauber 2017). The synergy between these forces created a powerful worldview that was inevitably projected onto the emerging science of the body’s defenses.

The Westphalian model of the sovereign state, with its emphasis on territorial integrity, clearly defined borders, and a sharp distinction between citizen and foreigner, provided the primary political metaphor. The body became a nation, a corporeal self-state. The work of Elie Metchnikoff and Paul Ehrlich, often cast as a debate between cellular and humoral immunity, can also be read as a discussion about the nature of the state’s military, whether its strength lay in a standing army (phagocytes) or in sophisticated weaponry (antibodies) (Tauber 2017). The discovery of the major histocompatibility complex (MHC) was seamlessly integrated into this narrative as the ultimate biological passport, the molecular marker of citizenship that distinguished self from the dangerous other (Pradeu 2012).

This political metaphor was supercharged by the rise of germ theory. The work of Louis Pasteur and Robert Koch shifted the medical gaze from internal imbalances (humoral theory) to external threats. The world was suddenly teeming with invisible enemies, microbial invaders that threatened to breach the borders of the body and cause disease. This created a perfect storm: the political concept of the foreign invader merged with the biological concept of the pathogenic microbe. As historian Warwick Anderson has noted, the language used to describe bacteriological threats was often indistinguishable from the language of colonial administration, which was preoccupied with sanitation, quarantine, and the control of contaminated native populations (Anderson 2006). The fear of the microbe and the fear of the colonized others were mutually reinforcing (Fanon 2008).

The anthropology of biomedicine has long documented this mirroring effect. Scholars such as Ed Cohen have traced how the legal-political concept of immunity, originally referring to an individual’s exemption from civic duties in Roman law, was mapped onto the biological body specifically during an era when the defense of borders became a primary geopolitical concern (Cohen 2009). Similarly, A. David Napier highlights how immunological dualisms (self-versus non-self) inherently reflect a Western philosophical preference for rigid boundaries and antagonistic relationships over integration and transition (Napier 2003).

This logic is a cornerstone of what Achille Mbembe terms necropolitics, the power of the sovereign to dictate who may live and who must die (Mbembe 2019). Colonialism was the ultimate necropolitical project, predicated on a racialized hierarchy that designated certain populations as disposable. It functioned through a logic of separation and purification, aiming to protect the purity of the colonizer from the contamination of the colonized (Anzaldúa 1987). This logic resonates with uncanny precision in the immunological concept of a pure self that must be defended at all costs from the non-self.

It is critical here to articulate the scope of this decolonial claim. We are not asserting a deterministic causal influence wherein colonial administrators directly dictated laboratory findings, nor are we claiming that the biological phenomenon of immunity is structurally equivalent to necropolitics. The immune system is not literally a fascist state. The immune system, in this telling, becomes the state’s biopolitical agent, enforcing a policy of purification by identifying and annihilating anything deemed foreign. The self/non-self-binary, the central dogma of classical immunology, is thus revealed not as an objective biological fact but as a historical heuristic heavily influenced by the colonial binary of colonizer/colonized, pure/impure, civilized/savage (Quijano 2000; Fanon 2008). These paradigms represent profound rhetorical resonances rather than strict structural identities; nonetheless, the metaphors chosen to constrain the scientific imagination, rendering certain biological realities visible while obscuring others.

This framework is so powerful because it naturalizes political order. It makes the nation-state, with its guarded borders and suspicion of the foreign, seem like a biological inevitability. But modern biology tells a different story.

### The Limits of Militarized Biology

For decades, the war metaphor served as a productive, if imperfect, framework. However, the conceptual revolutions of recent biology have stretched it to its breaking point. Three areas in particular reveal their profound inadequacy: the holobiont, immune tolerance, and the nature of chronic disease.

First, the concept of the holobiont shatters the very foundation of the sovereign self (Gilbert et al. 2012). The recognition that our bodies are co-constituted by trillions of bacteria, viruses, fungi, and archaea, our microbiota transforms the biological individual from a singular entity into a multi-species community (Yong 2016). We are not a self but a we, an ecological composite whose metabolic, neurological, and immune functions are deeply intertwined with our microbial partners. From a military perspective, the holobiont is an absurdity. It would mean that the sovereign state is, in fact, mostly populated by foreigners who are somehow essential for the states functioning. The war metaphor forces us to describe our relationship with our microbiome in the tense language of occupation, containment, and surveillance, a begrudgingly tolerated fifth column, as it were. This fails to capture the reality of symbiosis, a dynamic and cooperative partnership that is fundamental to our health.

Second, the war metaphor renders immune tolerance paradoxical. Tolerance is the active, ongoing process by which our immune system learns and maintains non-reactivity to our own tissues and to our symbiotic microbial partners. It is the default grammar of health and coexistence. Yet, within a military framework, tolerance can only be understood as a failure to attack, a dangerous lapse in vigilance (Pradeu 2012). Autoimmunity, the breakdown of self-tolerance, is then framed as a civil war or friendly fire, a language that assigns malicious intent or tragic error to our own cells. This perspective obscures the reality that tolerance is not a passive state but a continuously negotiated and highly regulated dialogue. The immune system is not primarily designed to attack, but to discern when not to attack.

Third, the military narrative falls short in explaining the most pressing health challenges of our time: chronic, non-communicable diseases. The war metaphor is best suited for acute infections, a clear enemy, a decisive battle, and a return to peace. It struggles to account for conditions like chronic inflammation, allergies, and many autoimmune diseases. Allergies are dismissed as overreactions or false alarms, casting the immune system as a paranoid general who mistakes a harmless diplomat (pollen) for an invading army. Chronic inflammation becomes a war of attrition or a smoldering fire, a chaotic and destructive process to be suppressed at all costs. This language prevents us from seeing these conditions for what they are more likely: not errors of war, but sustained states of systemic disequilibrium, often resulting from a mismatch between our evolutionary biology and our modern environment (Gluckman and Hanson 2006; Lea et al. 2023). The hygiene hypothesis, for instance, suggests that a lack of early and diverse microbial exposure or biota alteration leaves our immune system uneducated and prone to crises of social anxiety (Parker et al. 2024).

The limitations are clear. The language of war flattens the complex, relational, and ecological reality of the immune system into a simplistic and brutal binary (Martin 1994). It is time for a new lexicon.

### A Decolonial Lexicon

To decolonize our understanding, we must replace the conceptual scaffolding. If the body is not a sovereign nation, what is it? I propose the body as a quilombo: a vibrant, diverse, multi-species community forged in a context of negotiation and the pursuit of equilibrium. Within this framework, the immune system is the community’s system of governance, communication, and diplomacy. Its primary role is not war, but the art of living together. This shift in metaphor allows us to remap the entire landscape of immunology.

It is important to position this proposed lexicon within the existing body of reformulations. Thomas Pradeu effectively argued for a continuity theory, substituting the self/non-self-binary with a threshold of biochemical discontinuity (Pradeu 2012). Donna Haraway has utilized cyborg figurations to break down naturalized bodily borders (Haraway 1991), while Gilbert and Sapp champion a symbiotic view of life (Gilbert et al. 2012). Where these frameworks excel in highlighting biochemical triggers and ecological entanglements, the quilombo metaphor specifically contributes to a sociopolitical dimension of negotiated governance and historical resilience. However, we must proceed with methodological caution. All metaphors possess limits. While quilombo highlights dialogue and community cohesion, it risks anthropomorphizing biochemical cascades granting cells conscious intentionality they do not possess or unintentionally romanticizing historical communities that were forged under the severe duress of enslavement. The pedagogical utility of the metaphor must remain distinct from the empirical mechanism.

To ensure this distinction, the following sections will explicitly define the biological mechanism in standard scientific terms before translating it into the proposed civic metaphor. The anatomy of the immune system can be reimagined, moving from military infrastructure to civic architecture.

#### Bone Marrow as the Community Nursery

Biologically, the bone marrow is the primary lymphoid organ where hematopoiesis occurs. Multipotent hematopoietic stem cells differentiate into various myeloid and lymphoid lineages, including erythrocytes, platelets, innate immune cells (macrophages, neutrophils, dendritic cells), and B-lymphocytes. Metaphorically, it is not a military recruitment center churning out soldiers for a single purpose. It is the generative heart of the community, a protected nursery where the immense diversity of the body’s citizens is conceived. From this creative core emerge not just pre-programmed fighters, but a vast range of future specialists: sanitation workers (phagocytes), long-distance communicators (lymphocytes), historians (memory cells), diplomats (regulatory cells), and transporters who keep the community nourished. It is the cradle of civic potential, the promise of a complex and multifaceted society.

#### Thymus as the University of Coexistence

In canonical immunology, the thymus is where T-cell precursors migrate to undergo somatic recombination of T-cell receptors (TCRs). This organ enforces central tolerance through two stages: positive selection (survival of T-cells capable of binding self-MHC molecules with moderate affinity) and negative selection (apoptosis of T-cells that bind self-antigens too strongly), ensuring MHC restriction and preventing autoimmunity. This is not an elite military academy for brutal training. It is a sophisticated school of philosophy and sociology, where potential T-lymphocytes undergo an education in relational literacy. The curriculum has two core disciplines. The first, positive selection, is a fundamental linguistics course where students learn to read the basic language of the community, the MHC molecules. Failure to learn this language renders a student incapable of participating in community dialogue. The second, negative selection, is an advanced course in sociology and tolerance. Here, students are introduced to the vast library of the community’s own components. If a student reacts with aggression, they are not seen as a traitor to be summarily executed. They are understood as an individual whose disposition is incompatible with communal harmony and are gently guided into a process of silent, non-inflammatory recycling: apoptosis (Abbas et al. 2019). Thymus is a graduating institution that ensures only those citizens are best suited for complex dialogue and peaceful coexistence proceed to public spaces.

#### Secondary Lymphoid Organs as Vibrant Civic Spaces

The sites where immune responses are mounted are not forts or forward operating bases, but the bustling civic centers of the community.

#### Lymph Nodes as Community Market Squares

Scattered throughout the body, these are not garrisons. They are marketplaces for information. Biologically, these encapsulate networks where afferent lymphatic vessels deliver lymph containing tissue-derived antigens and antigen-presenting cells (APCs). Within the node, APCs interact with naive T and B lymphocytes in defined zones to initiate adaptive immunity. The lymph fluid does not transport prisoners of war, but travelers and messengers carrying news and product samples (antigens) from the peripheral regions. It is in these squares that diplomats, historians, and stewards meet to discuss the news and decide if new management policies are needed. A swollen lymph node is not a besieged fort; it is a central square packed with citizens during an urgent community assembly.

#### Spleen as the National Library and Recycling Center

Monitoring the body’s main communication highway (the blood), the spleen serves a dual function. It filters blood-borne antigens in the white pulp to stimulate adaptive immune responses, while macrophages in the red pulp phagocytose aged or damaged erythrocytes. It is a vast historical archive, housing experienced citizens (memory cells) who hold the records of past encounters. It is also a sanitation center that, with dignity, removes and recycles the community’s oldest members, like senescent red blood cells, repurposing their components for the common good. It is a center of wisdom and sustainability, not a military headquarters (Murphy and Weaver 2017).

#### MALT (Mucosa-Associated Lymphoid Tissue) as the Port of Cultural Exchange

Nowhere is the inadequacy of the war metaphor more evident than at the mucosal surfaces. Mechanistically, MALT comprises non-encapsulated lymphoid clusters that continuously sample luminal antigens via specialized M cells. Here, regulatory T-cells (Tregs) and tolerogenic dendritic cells heavily bias the response toward the production of secretory IgA and active immune tolerance towards symbiont microbiota. The gut, our primary interface with the world, is not a fortified border wall. It is the most cosmopolitan of spaces: a bustling port in constant dialogue with an immense and diverse population of partners (the microbiota) and strangers (food antigens) (Yong 2016). Its primary function is not exclusion, but negotiation, tolerance, and learning. It is here that the community’s most experienced diplomats reside, masters in the art of distinguishing a commercial partner from a subversive agent. The MALT is proof that our identity is forged in relation, not in isolation (Gilbert et al. 2012).

With this new civic geography, we can re-describe the dynamic processes of immunity not as battles, but as sophisticated forms of communication and self-organization.

#### Inflammation as an Urgent Community Assembly

When the community faces perturbation, structural damage like a cut or the presence of a subversive agent, its immediate response is inflammation. In standard biological terms, acute inflammation is initiated when tissue-resident macrophages and mast cells detect damage-associated molecular patterns (DAMPs) or pathogen-associated molecular patterns (PAMPs) via pattern recognition receptors (PRRs). These cells release vasoactive amines (like histamine) and pro-inflammatory cytokines (like TNF and IL-1), inducing vasodilation, increasing vascular permeability, and promoting the chemotaxis of leukocytes to the affected site.

In the colonial narrative, this is the fire of war, a chaotic and destructive event to be suppressed. In our relational reading, serving as a descriptive heuristic, inflammation is an Urgent and Heated Community Assembly. It is the orderly mechanism by which the community gathers to deliberate on a problem, execute a solution, and initiate healing (Medzhitov 2008). Its famous cardinal signs are not signs of chaos, but of a highly organized civic mobilization.

#### Heat and Redness

The first signaling molecules cause local vasodilation. The goal is not to send in troops haphazardly, but to increase the flow of communication and resources. It is the equivalent of opening a city’s main avenues to allow more citizen-cells, mediation specialists, and logistical resources to reach the site of the disturbance quickly.

#### Swelling/Edema

The increased permeability of blood vessels allows for the formation of a physical space for the debate. This is not the chaotic extravasation of fluids onto a muddy battlefield. It is the creation of a true assembly hall. Not only do cells arrive, but also the support material: documents and memoranda (antibodies and complement proteins) and plasma itself, creating an environment where participants can move, interact, and dialogue freely.

#### Dolor (Pain)

Mobilization communicates with the organism’s central consciousness through pain. Pain is not a simple cry from wounded tissue; it is one of the body’s most sophisticated communication signals. It is a request for guardianship and mindful attention. The local community sends a clear message to the nervous system: Attention, we are conducting a delicate and important assembly here. Please protect this area. Do not overburden it with other tasks.

#### Functio Laesa (Loss of Function)

Often seen as inevitable collateral damage, this can be reinterpreted as a deliberate and programmed act of governance. The community decides to temporarily suspend normal activities in that neighborhood so that the assembly, cleanup, and subsequent reconstruction can proceed in an orderly fashion. It is akin to closing a street for infrastructure repairs, a sign not of destruction, but of a well-managed and protected healing process.

This perspective also allows us to differentiate between types of inflammation. Acute inflammation is the community assembly as it should be, intense, focused, and with a clear purpose and a mechanism for conclusion. Once the problem is resolved, a reconstruction committee takes over, and the community returns to its routine (Serhan 2010). Chronic inflammation, by contrast, is the assembly that never ends. It is a low-volume debate with no clear agenda and no effective moderation; a hum of social anxiety that slowly depletes resources and degrades the very structure of the tissue.

#### The Adaptive Response as a Journey of Information

When a local disturbance exceeds the management capacity of the local stewards, the community does not escalate to total war. It initiates one of biology’s most elegant processes: a flow of information, deliberation, and learning. The adaptive response is not about creating weapons but about transforming a local news story into enduring historical wisdom (Murphy and Weaver 2017).

To map the underlying mechanism accurately: this process commences when an antigen-presenting cell (APC), such as a dendritic cell, captures an antigen in peripheral tissues via phagocytosis. The APC processes the antigen into peptide fragments, loads them onto MHC class II molecules, and migrates to the draining lymph node. There, it presents the MHC-peptide complex to naive CD4 + T-cells. Activation requires two specific signals: the binding of the T-cell receptor (TCR) to the MHC-peptide complex (Signal 1), and a co-stimulatory interaction, such as CD80/86 on the APC binding to CD28 on the T-cell (Signal 2). Once activated, T-cells secrete cytokines to direct other cells, including B-cells, which, after binding their specific antigen and receiving T-cell help, differentiate into antibody-secreting plasma cells or long-lived memory cells.

Translated into our civic metaphor, it begins with a dedicated Investigative Journalist, a dendritic cell, patrolling a peripheral neighborhood like the skin. There, it finds a cluea fragment of a subversive agent or a protein from a damaged cell. Its job is not to attack, but to investigate. It gathers the raw material of the story (phagocytosis) and, inside, begins an editorial process, breaking down the complex narrative into short, clear headlines: peptides.

With its edited report, the journalist undertakes a journey to the nearest community center, the lymph node. There, it steps onto a public pulpit, the MHC molecule, and publishes its headline for qualified citizens to evaluate. Circulating constantly through these centers are the diplomats and sociologists of the community, the T-lymphocytes. When a T-cell whose perception is perfectly compatible with the presented headline finds it, a specific recognition occurs. But to prevent the spread of rumors, the diplomat requires confirmation. It establishes a dialogue with the journalist, asking if the news is truly relevant. If the perturbation was genuine, the journalist expresses a sign of concordance, a molecular handshake known as co-stimulation. This confirmation transforms a simple reading into a serious conversation (Abbas et al. 2019).

With the news validated, the T-lymphocyte transforms from a passive listener into an active opinion leader. It forms a committee of specialists on that story (clonal expansion) and begins to issue directives and opinions, cytokines that will orchestrate the community’s response.

Meanwhile, *the historians and archivists of the community*,* the B-lymphocytes*, may have also found clues directly. Before mass-producing a record, however, they seek editorial validation. They meet with an activated T-cell and present the same headline. This cross-dialogue is a double-checking system of remarkable elegance, ensuring that only information validated by two independent branches of the community’s intelligence agency becomes official policy (Murphy and Weaver 2017).

Once authorized, the B-cell historian undergoes a dramatic transformation into a high-production printing press, a plasma cell. Its entire energy is dedicated to producing and releasing millions of copies of a single document: the official memorandum, the antibody. Distributed through all circulation routes, this memo carries clear instructions for managing that specific perturbation throughout the community.

The community, in its wisdom, knows this perturbation may recur. It would be a waste of energy to repeat this entire slow process of journalism and deliberation. Therefore, a portion of the T and B cells that participated in the debate are given a new, honorable function: they become veteran historians and guardians of the archive, memory cells. They persist for years, constituting the living memory of the community. The next time the same news story appears; these experienced veterans will recognize it instantly. The long process of confirmation will be unnecessary. The response will be immeasurably faster and more robust. This is how the community learns: not through brute force, but through a process of journalism, debate, validation, mass publication, and, most importantly, the creation of a historical memory that makes it wiser and more resilient for future encounters (Tauber 2017).

#### Crises of Identity and Social Misunderstanding

This relational paradigm provides a more compassionate and biologically plausible lens for pathology, reframing diseases from military failures to complex and tragic social crises. Once again, i emphasize that these are alternative heuristic framings designed to replace counterproductive idioms like friendly fire, rather than precise descriptions of pathogenesis.

#### Allergy and the Hygiene Hypothesis

An allergy can be understood as a cultural misunderstanding or a crisis of social anxiety. This aligns elegantly with the hygiene or mismatch hypothesis (Gluckman and Hanson 2006). A community that develops in a super-hygienic environment, with little exposure to a rich diversity of microbes, can be compared to an individual who has not had the necessary intercultural education (Parker et al. 2024). It has not been learned to deal with a variety of strangers with calm and moderation. Thus, when it encounters an unknown but harmless entity like pollen, its reaction is not a calculation error, but a panic attack.

#### Autoimmunity

The most profound tragedy: a conflict turned inward. The language of civil war or treason is deeply problematic, as it attributes intent and blame to the community’s own cells. A decolonial, relational view suggests a different narrative: a crisis of community identity or an unresolved historical trauma. The social fabric has been torn. This can happen through semantic confusion (molecular mimicry), where a subversive agent possessed a molecular signature so like a citizen that the legitimate defensive response continues, by a tragic linguistic mistake, against the citizen itself (Abbas et al. 2019). In other scenarios, the crisis can emerge from persistent trauma that leaves the community in a state of post-traumatic stress, where stewards and diplomats remain on high alert, perpetuating a cycle of damage. Perhaps most importantly, the crisis can arise from failure in the regulatory network. The council of elders, the regulatory T-cells that preach calm and memory may have been silenced or lost its authority. Without these voices of moderation, small internal disputes escalate into destructive conflicts that threaten the community’s integrity.

#### Cancer and Transplantation

These phenomena represent the outer limits of community resilience. The military narrative frames them simply: cancer is an internal insurrection, and a transplanted organ is a foreign invasion. This view obscures their relational complexity. I propose seeing them, respectively, as a crisis of social alienation and an attempt at intercultural fusion of the highest complexity. Cancer is not a deliberate betrayal but the result of a profound process of alienation. Tumor cells are members of the community that, due to accumulated damage, have forgotten the language of coexistence. They stop listening to their neighbors, ignoring signals to limit growth. Immune surveillance, in this context, is not the action of secret police hunting traitors, but the work of conflict mediators and social stewards trying to reintegrate or silently remove individuals who have lost their sense of belonging. Immunotherapy can be seen not as arming the police, but as a large-scale social re-education intervention. Transplant rejection, conversely, is not xenophobia. It is a violent and catastrophic cultural clash. The identity languages (MHC molecules) of the two communities are so distinct that dialogue becomes impossible, resulting in a gigantic misunderstanding that escalates into a destructive conflict (Murphy and Weaver 2017). Immunosuppressive therapy is therefore not disarming the army, but the imposition of a mediated silence to facilitate intercultural learning and allow a new, hybrid culture to emerge (Haraway 1991).

Immunodeficiency states are not signs of a weak army but of failure in civic infrastructure. Primary immunodeficiencies are developmental challenges, where the community is born with a structural difficulty in performing a social function. A defect in antibody production is a crisis in the ability to build and maintain a historical archive. A severe T-cell deficiency represents a crisis in the University of Coexistence and the Diplomatic Corps. Acquired immunodeficiencies, like AIDS, represent the collapse of social cohesion in a previously functional community. The HIV virus is not a military saboteur but an agent that dismantles the central hubs of political dialogue (CD4 + T-cells), causing a blackout of diplomatic wisdom that leaves the community vulnerable (Abbas et al. 2019).

## Conclusion

Our journey has guided us through a new geography of the body, trading the map of war for the landscape of a vibrant community. We have reinterpreted inflammation not as the fire of battle, but as a heated community assembly. We have followed the flow of information from an investigative report to its inscription in the archives of historical memory. Throughout, the proposal has been simple yet profound: to shift our gaze from violence to relation, from conflict to dialogue.

This re-framing, however, is not intended as an intellectual exercise or a collection of new metaphors, nor is it presented as a definitive corrective to modern biomedical empiricism. Its purpose is primarily pedagogical and, ultimately, ethical. The way we teach science is not a neutral act of transmitting facts; it is the sharing of a worldview. The bellicose language of immunology, inherited from an era of imperialism and consolidated in a world of nation-states, trains professionals to see the body as a battlefield, disease as an enemy to be annihilated, and medicine as a military intervention (Sontag 2001; Martin 1994). While this perspective may have value in acute situations, it severely limits our ability to comprehend the complexity of chronic diseases, the importance of the body as an ecosystem, and health as a process of dynamic equilibrium.

To offer an immunology of encounters, therefore, is an invitation to form a new generation of health and science professionals. A generation that, in learning about the immune system, also learns about communication, interdependence, and the wisdom of living systems. A professional who understands chronic inflammation as low-level social anxiety may be better equipped to investigate systemic lifestyle causes rather than simply prescribing a drug to silence the alarm. A student who sees the holobiont as a multi-species community can develop a deeper respect for the complex ecology of their future patients and the planet (Yong 2016). To teach immunology as the management of relations is to cultivate an ethic of care, of listening, and of the search for harmony, rather than an ethic of conquest.

Perhaps the greatest value of this perspective shift is its capacity to connect us to broader questions. The logic that sees a virus only as an invader to be eradicated is not so different from the logic that sees a forest as a resource to be exploited, or a different culture as a threat to be contained (Krenak 2019). Both are born from a worldview that privileges separation, purity, and control (Haraway 1991). By relearning to see our own bodies as spaces of encounter, negotiation, and coexistence, we might take a small step toward relearning how to inhabit the world in the same way.

This paper does not offer easy answers or a definitive protocol. It is a proposal, an opening, an attempt to build a bridge between cutting-edge science and a decolonial critique of modernity (Viveiros de Castro 2017). It is a call for teachers and students to become active participants in the construction of knowledge, questioning the narratives we have inherited and daring to imagine others. The task is immense, for it implies decolonizing not only our curricula, but our own minds (Quijano 2000; Fanon 2008). The task is to retexture the world. And that patient, deliberate work can begin here, in the fascinating universe of our own body, as we learn to listen to the intricate and resilient conversation of life.

## Data Availability

No datasets were generated or analysed during the current study.
